# Serum Fetuin-A Associates with Type 2 Diabetes and Insulin Resistance in Chinese Adults

**DOI:** 10.1371/journal.pone.0019228

**Published:** 2011-04-27

**Authors:** Aiyun Song, Min Xu, Yufang Bi, Yu Xu, Yun Huang, Mian Li, Tiange Wang, Yaohua Wu, Yu Liu, Xiaoying Li, Yuhong Chen, Weiqing Wang, Guang Ning

**Affiliations:** 1 Shanghai Clinical Center for Endocrine and Metabolic Diseases, Shanghai Institute of Endocrine and Metabolic Diseases, Department of Endocrinology and Metabolism, Ruijin Hospital Affiliated to Shanghai Jiao Tong University School of Medicine, Shanghai, China; 2 State Key Laboratory of Medical Genomics, Ruijin Hospital Affiliated to Shanghai Jiao Tong University School of Medicine, Shanghai, China; University of Hong Kong, China

## Abstract

**Background:**

Previous studies have demonstrated that fetuin-A is related to insulin resistance among subjects with normal glucose tolerance but not patients with type 2 diabetes. There are limited data available concerning fetuin-A and insulin resistance in Chinese. We aimed to study the association of feuin-A with insulin resistance among participants with or without type 2 diabetes in a large sample size of adults aged 40 and older.

**Methodology and Principal Findings:**

A community-based cross-sectional study was performed among 5,227 Chinese adults. The average age of our study was 61.5±9.9 years. Serum fetuin-A concentrations were not significantly different between male and female (296.9 vs. 292.9 mg/l, p = 0.11). Compared with the lowest quartile, the highest quartile of serum fetuin-A revealed a significant higher proportion of type 2 diabetic patients (34.8% vs. 27.3%, p<0.0001). In the multinomial logit models, the risk of type 2 diabetes was associated with each one quartile increase of serum fetuin-A concentrations when referenced not only to normal glucose tolerance (OR 1.24, 95% CI 1.07–1.43, p = 0.004) but also to impaired glucose regulation (OR 1.25, 95% CI 1.08–1.44, p = 0.003, respectively), after adjustment for age, sex, community, current smoking, and current drinking. The logistic regression analysis showed that fetuin-A were associated with elevated HOMA-IR and fasting serum insulin both among the participants with or without type 2 diabetes in the full adjusted analysis. There was no significant association between elevated serum fetuin-A concentrations and impaired glucose regulation (all p≥0.12).

**Conclusions and Significance:**

Higher fetuin-A concentrations were associated with type 2 diabetes and insulin resistance in middle aged and elderly Chinese.

## Introduction

Serum fetuin-A (also called alpha-2 heremans schmid glycoprotein, AHSG) is a multifunctional glycoprotein which is exclusively secreted from hepatocytes in human [1]. For a long time, fetuin-A has been considered to play a crucial role in the protection from vascular calcification by solubilizing calcium and phosphorus in serum [2]–[5]. It was also reported that fetuin-A could inhibit insulin receptor tyrosine kinase activity through blocking the autophosphorylation of tyrosine kinase and insulin receptor substrate-1(IRS-1), and induced a lower-grade inflammation [6], [7], which resulted in insulin resistance [8]–[11]. Recently, epidemiological studies showed that serum fetuin-A was associated with insulin resistance [12], [13] and its co-morbidities, such as metabolic syndrome [14], [15] and type 2 diabetes [16], [17].

Impaired glucose regulation (IGR), also termed as prediabetes consisting of impaired fasting glucose and/or impaired glucose tolerance (IFG and/or IGT) is definitely a risk factor for type 2 diabetes and cardiovascular disease [18], [19]. The prediabetes is abruptly increased in the world, as well as in China [20]. Insulin resistance is a conspicuous characteristic of prediabetic states. Thus, it is indispensable to evaluate the association of serum fetuin-A with type 2 diabetes, as well as with prediabetes.

In the present study, we investigated the association of serum fetuin-A with prediabetes, type 2 diabetes and insulin resistance in a community-based Chinese population aged 40 or above.

## Methods

### Ethics statement

This study was approved by the Institutional Review Board of the Ruijin Hospital, Shanghai Jiao Tong University School of Medicine and was in accordance with the principle of the Helsinki Declaration II. The written informed consent was obtained from each participant.

### Study population

The participants were recruited from an ongoing glucose survey in Youyi and Songnan communities, Baoshan, Shanghai in 2005 and 2008, respectively. The study population, design and protocols of the study have been previously described [21], [22]. In brief, we first invited all registered permanent residents aged 40 or above by poster advertisement and by mail to participate in a screening examination. The screened subjects were allocated into 3 subgroups according to their fasting glucose concentrations of 7.0 mmol/l or above, 5.6 to 6.9 mmol/l and below 5.6 mmol/l. We then selected randomly a case-control cohort from these 3 subgroups for further investigation, including a simplified 75-g oral glucose tolerance test (OGTT), fasting and 2 hour blood and urine sampling. We intended to compensate the potential low participation rate of those with lower glucose levels by a defined ratio of 1.0∶1.2∶1.4 in the above subgroups. The total 5847 subjects were eventually allocated into normal glucose tolerance (NGT), IGR and type 2 diabetes based on OGTT results. The participants and non-participants were similar in characteristics, such as age and sex distribution. The following subjects were excluded in the final analysis, including those with more than five times of the normal serum alanine aminotransferance (ALT), aspartate aminotransferance (AST), or γ-glutamyl transpeptadase (GGT) levels, or with less than 30 ml/min/1.73 m^2^ of the estimated glomerular filtration rate (eGFR), or with self-reported fatty liver disease, or missing serum fetuin-A concentrations, or with body mass index (BMI) less than 18.5 kg/m^2^ or great than 40 kg/m^2^. Thus, a total of 5227 subjects, including 2008 NGT, 1621 IGR and 1598 type 2 diabetic patients were included in the final analysis.

### Clinical and biochemical measurements

Body height, body weight, and waist and hip circumferences were measured by the experienced physicians. Blood pressure was measured at the non-dominant arm in a seated position after a ten-min rest using an automated electronic device (OMRON Model HEM-752 FUZZY, Omron Company, Dalian, China). Three measurements were taken in one minute apart and the average of the three measurements was used in analysis.

All participants underwent OGTT after an overnight fast. Fasting and 2 hour plasma glucose concentrations were measured within one hour using the glucose oxidase method on an autoanalyser (ADVIA-1650 Chemistry System, Bayer Corporation, Germany). Fasting serum insulin concentrations were measured using an electrochemiluminescence assay (Roche-Diagnostics, Switzerland). Fasting serum ALT, AST, GGT, albumin, bilirubin, creatinine (SCR), triglycerides (TG), total cholesterol (TC), high density lipoprotein cholesterol (HDL-C), low density lipoprotein cholesterol (LDL-C) and high sensitivity C reactive protein (hs-CRP) were measured using an autoanalyser (ADVIA-1650 Chemistry System, Bayer Corporation, Germany).

Fasting serum fetuin-A concentrations were measured using a human fetuin-A sandwich Enzyme Linked-Immuno-Sorbent assay kit (MAB1184 R&D Company, CA, USA.). The inter and intra assay coefficient of variation is 5.2% and 7.8%, respectively.

### Definitions

Type 2 diabetes was diagnosed according to the 1999 World Health Organization (WHO) criteria (fasting plasma glucose level ≥7.0 mmol/l and/or 2-h OGTT plasma glucose level ≥11.1 mmol/l, or taking anti-diabetic agents). IGR was defined as IFG (fasting plasma glucose level ≥6.1 and <7.0 mmol/l) and/or IGT (2 h OGTT plasma glucose level ≥7.8 and <11.1 mmol/l). Insulin resistance index (homeostasis model assessment of insulin resistance, HOMA-IR) was calculated using homeostasis model assessment methods, as fasting insulin (IU/ml)×fasting glucose (mmol/L)/22.5. The abbreviated Modification of Diet in Renal Disease (MDRD) formula recalibrated for Chinese [23] was used to calculate the eGFR expressed in mL/min per 1.73 m^2^: eGFR = 186×[SCR×0.011]−1.154×[age]−0.203×[0.742 if female]×1.233, where SCR is serum creatinine expressed as µmol/L and 1.233 is the adjusting coefficient for Chinese. BMI was calculated as weight in kilometers divided by height in meters squared. Waist-to-hip ratio (WHR) was calculated as waist divided by hip circumference in the same unit.

### Statistical analysis

SAS version 8.1 (SAS Institute, Cary, NC) was used for database management and statistical analysis. Measurements with a skewed distribution, serum fetuin-A, TG, AST, ALT, GGT, CRP and HOMA-IR were normalized by logarithmic transformation. Comparisons of means and proportions were performed with the standard normal z-test and x^2^ tests, respectively. To allow for covariates and confounders, we performed analysis of covariance and multiple linear and logistic regressions. Pearson correlation analysis and multiple linear regression analysis were performed to evaluate the association between serum fetuin-A and the risk factors of type 2 diabetes. In order to eliminate the effect of antidiabetic treatment on serum insulin and plasma glucose levels as well as HOMA-IR, we performed the simple correlation and multiple linear regression analysis by excluding the subjects who were receiving oral antidiabetic treatment or insulin injection (n = 611). The subjects were also categorized into quartiles of serum fetuin-A concentrations. Quartile 1 represents the lowest and quartile 4 represents the highest one. We used the multinomial logit analysis to evaluate the association of serum fetuin-A with type 2 diabetes and IGR. The multivariate logistic regression analysis was used to study the association of serum fetuin-A with higher levels of HOMA-IR, fasting serum insulin. In our study, subjects with equal to and/or more than the highest quartile of HOMA-IR index, 2.18 in non-type 2 diabetes group and 4.64 in type 2 diabetes group were defined as having insulin resistance. Subjects with equal to and/or more than the highest quartile of fasting serum insulin, 9.4 µIU/ml in non-type 2 diabetes group and 13.9 µIU/ml in type 2 diabetes were defined as having elevated levels of fasting serum insulin.

## Results

The distribution of serum Fetuin-A concentrations was positively skewed with a median of 294.9 mg/l (inter-quartile range 234.4–366.4 mg/l). Characteristics of study participants among groups of NGT, Iso-IFG, Iso-IGT, IFG and IGT, and type 2 diabetes were shown in Table 1. The average age of our study, including 2051 (39.2%) male and 3176 (60.8%) female, was 61.5±9.9 years. Serum fetuin-A concentrations were not significantly different between male and female (296.9 vs. 292.9 mg/l, p = 0.11). Serum fetuin-A concentrations in type 2 diabetic patients were significantly higher than those with NGT or IGR, after adjustment for age, sex and community (p = 0.0008). However, serum fetuin-A concentrations were not significantly different between the participants with NGT and those with IGR and among all sub-groups with IGR, including Iso-IFG, Iso-IGT, IFG and IGT group (Iso-IFG vs Iso-IGT, 290.7 vs 290.5 mg/l, P = 0.46; Iso-IFG vs IFG and IGT, 290.7 vs 291.0 mg/l, p = 0.95; Iso-IGT vs IFG and IGT, 290.5 vs 291.0 mg/l, p = 0.79) Among 1598 type 2 diabetic patients, 611 were given the treatment of oral antidiabetic drugs or insulin injection. Among them, 575 patients were given oral antidiabetic drugs and 84 were insulin injection and 48 were the both therapies. Fetuin-A concentrations were not significantly different between patients with and without therapy (301.8 vs 311.6 mg/l, p = 0.42). The presences of type 2 diabetes across fetuin-A quartile groups were 27.3%, 26.6%, 33.6%, and 34.8%, respectively. The presence of type 2 diabetes in the highest fetuin-A quartile was higher than that in the lowest quartile (p<0.0001). The presences of IGR across fetuin-A quartile groups were 32.5%, 31.9%, 30.0%, 29.7%, respectively (p = 0.07).

**Table 1:** Characteristics of the study population.

	Normal glucose tolerance	Impaired Glucose Regulation			Type 2 diabetes	p-value
		Isolated IFG	Isolated IGT	IFG and IGT		
Number	2008	515	602	504	1598	
Demographics						
Age (years)	59.3±9.6	60.4±9.3	62.4±10.2	62.9±9.3	63.8±9.7	<0.0001
Male, n (%)	711 (35.4)	218 (42.3)	229 (38.0)	188 (37.3)	705 (44.1)	0.0019
Current smoking, n (%)	420 (20.9)	89 (17.3)	110 (18.3)	67 (13.3)	314 (19.7)	0.9893
Current drinking, n (%)	281 (14.0)	76 (14.8)	100 (16.6)	66 (13.1)	245 (15.3)	0.7803
Measurements						
Body mass index (kg/m^2^)	24.5±3.1	24.9±3.1	25.7±3.3	25.8±3.1	26.3±3.6	<0.0001
Waist/hip ratio	0.88±0.06	0.88±0.06	0.91±0.06	0.90±0.06	0.92±0.06	<0.0001
Systolic blood pressure (mmHg)	132±21	136±22	142±22	143±22	147±22	<0.0001
Diastolic blood pressure (mmHg)	77±10	79±10	80±10	81±10	81±11	<0.0001
Fasting plasma glucose (mmol/L)	4.9±0.4	5.9±0.3	4.9±0.4	6.0±0.4	7.6±2.4	<0.0001
2 h OGTT plasma glucose (mmol/L)	6.0±1.1	6.4±1.0	8.9±0.9	9.1±1.0	16.0±5.1	<0.0001
Fasting serum insulin (µIU/ml)	5.1 (4.9–5.2)	5.8 (5.5–6.2)	6.4 (6.1–6.8)	7.5 (7.0–8.0)	8.3 (8.0–8.6)	<0.0001
HOMA-IR	1.11 (1.07–1.14)	1.51 (1.41–1.62)	1.44 (1.35–1.53)	2.00 (1.87–2.14)	2.72 (2.62–2.82)	<0.0001
Total cholesterol (mmol/l)	4.99±0.91	5.00±0.88	5.14±0.89	5.10±0.99	5.17±1.11	<0.0001
Triglycerides (mmol/l)	1.22 (1.19–1.25)	1.36 (1.29–1.43)	1.48 (1.42–1.55)	1.65 (1.56–1.73)	1.76 (1.71–1.81)	<0.0001
HDL-cholesterol (mmol/l)	1.42±0.34	1.43±0.33	1.36±0.33	1.36±0.33	1.31±0.32	<0.0001
LDL-cholesterol (mmol/l)	2.42±0.66	2.68±0.74	2.51±0.69	2.66±0.75	2.55±0.80	<0.0001
Glomerular filtration rate ml/min/1.73 m^2^	114.5±42.3	119.0±23.7	111.0±24.1	116.4±26.5	117.2±31.4	<0.0001
C-reactive protein (mg/l)	0.3 (0.3–0.4)	0.4 (0.4–0.5)	0.6 (0.5–0.7)	0.6 (0.5–0.7)	0.8 (0.7–0.9)	<0.0001
Fetuin-A (mg/l)	285.3 (231.9–359.1)	290.7 (226.9–372.9)	290.5 (234.5–359.2)	291.0 (230.0–359.9)	307.7 (243.5–376.2)	0.0008

Values are means ± SD or median (inter-quartile range) or number (proportion).

p-values were for the ANOVA across the five groups after adjustments for age, sex and community, or chi-square analysis.

Serum feuin-A concentrations were positively correlated with BMI, WHR, systolic and diastolic blood pressure, fasting serum insulin concentrations, fasting and 2 hour plasma glucose, HOMA-IR, serum TC, TG, and LDL-C (all p<0.007), and negatively correlated with HDL-C(r = −0.05, p = 0.001) The multivariate regression analysis showed that serum fetuin-A concentrations were independently correlated with diastolic blood pressure (β = 0.002, p = 0.006), fasting serum insulin concentrations (β = 0.03, p = 0.0009), HOMA-IR (β = 0.03, p = 0.0002) and serum TG (β = 0.05, p<0.0001). We also investigated the co-linearity between serum fetuin-A concentrations, BMI, WHR, systolic and diastolic blood pressure, fasting serum insulin concentrations, fasting and 2 hour plasma glucose, HOMA-IR, serum TC, TG, HDL-C and LDL-C, and CRP. We found all the variables were correlated between each other, but only the r value between HOMA-IR and fasting serum insulin was great than 0.80 (r = 0.97, p<0.0001).

The risk of IGR and type 2 diabetes in relation to quartiles of serum fetuin-A was analyzed by using multinomial logit models. Each one quartile increase of serum fetuin-A concentrations was significantly associated with increased risk of type 2 diabetes when referenced not only to NGT but also IGR (Model 2, OR 1.24, 95% CI 1.07–143, p = 0.004; OR 1.25 95% CI 1.08–1.44, p = 0.003, respectively), after adjustment for age, sex, community, current smoking, current drinking (Table 2). Referenced to IGR, when further adjusted for BMI and WHR, or blood lipids, or blood pressure, or CRP based on Model 2, the association between fetuin-A and type 2 diabetes did not radically changed; whereas referenced to NGT, the association between fetuin-A type 2 diabetes was remained after adjusted for blood pressure or CRP and attenuated after adjustment for BMI or WHR (p = 0.05 and 0.06, respectively). However, the associations disappeared when further adjusted for HOMA-IR referenced not only to NGT but also IGR (p = 0.68 and 0.14, respectively). When adjusted the most cofactors simultaneously, the association of fetuin-A and type 2 diabetes referenced to IGR still persisted (model 9, OR 1.21, 95% CI 1.04–1.40, p = 0.01). Even after excluding 611 subjects with antidiabetic treatment or insulin injection, the odds ratio of the risk of type 2 diabetes in relation to quartiles of serum fetuin-A concentrations increased when adjusted for age, sex, community, current smoking, current drinking, BMI, WHR, blood pressure, TC, logTG, HDL-C, LDL-C and logCRP (OR 1.19, 95% CI 1.01–1.40, p = 0.04). There was no significant association between elevated serum fetuin-A concentrations and IGR (all p≥0.12).

**Table 2:** The risk of impaired glucose regulation and type 2 diabetes in relation to quartiles of serum fetuin-A by using multinomial logit models.

	Impaired glucose regulation		Type 2 diabetes1		Type 2 diabetes2	
	ORs (95% CI)	p for trend	ORs (95% CI)	p for trend	ORs (95% CI)	p for trend
Model 1	0.97 (0.85–1.12)	0.72	1.26 (1.10–1.45)	0.001	1.30 (1.12–1.49)	0.0003
Model 2	0.99 (0.86–1.15)	0.93	1.24 (1.07–1.43)	0.004	1.25 (1.08–1.44)	0.003
Model 3	0.96 (0.83–1.11)	0.62	1.16 (1.00–1.34)	0.05	1.20 (1.04–1.40)	0.01
Model 4	0.96 (0.83–1.11)	0.57	1.15 (0.99–1.33)	0.06	1.20 (1.04–1.40)	0.01
Model 5	0.89 (0.77–1.03)	0.12	1.09 (0.94–1.26)	0.24	1.23 (1.06–1.42)	0.006
Model 6	0.98 (0.85–1.14)	0.82	1.23 (1.07–1.43)	0.005	1.25 (1.08–1.45)	0.002
Model 7	0.92 (0.79–1.07)	0.29	1.03 (0.89–1.20)	0.68	1.12 (0.96–1.30)	0.14
Model 8	0.96 (0.83–1.11)	0.56	1.20 (1.04–1.39)	0.01	1.25 (1.08–1.49)	0.002
Model 9	0.88 (0.75–1.02)	0.08	1.06 (0.91–1.23)	0.48	1.21 (1.04–1.40)	0.01

Data are odds ratios (ORs, 95% confidential interval).

Model 1, unadjusted;

Model 2, adjusted for age, sex, community, current smoking, and current drinking;

Model 3, based on model 2 further adjusted for BMI;

Model 4, based on model 2 further adjusted for WHR;

Model 5, based on model 2 further adjusted for blood lipids, including TC, logTG, HDL-C, LDL-C;

Model 6, based on model 2 further adjusted for blood pressure;

Model 7, based on model 2 further adjusted for logHOMA-IR;

Model 8, based on model 2 further adjusted for logCRP;

Model 9, based on model 2 further adjusted for BMI, WHR, blood pressure, TC, logTG, HDL-C, LDL-C, logCRP;

Type 2 diabetes^1^, referenced to normal glucose tolerance; Type 2 diabetes ^2^, referenced to impaired glucose regulation;

Odds ratios were calculated with the use of multinomial logit models.

**Figure 1:**
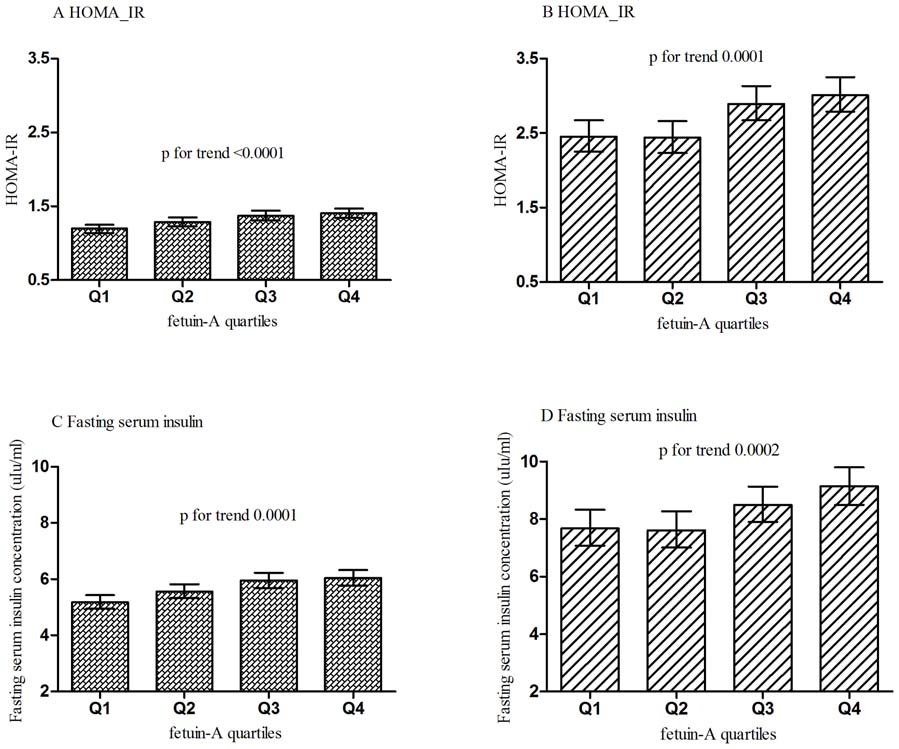
Levels of HOMA-IR and fasting serum insulin across quartiles of serum fetuin-A concentrations among non-type 2 diabetes and type 2 diabetes. (A): Levels of HOMA-IR among non-type 2 diabetes group; (B): Levels of HOMA-IR among type 2 diabetes group; (C): Levels of fasting serum insulin among non-type 2 diabetes; (D): Levels of fasting serum insulin among type 2 diabetes. Values are geometric means (95% confidential intervals) of HOMA-IR and fasting serum insulin. The adjusted variables included age, sex, community, BMI, current smoking, current drinking.

Among participants without type 2 diabetes, HOMA-IR and fasting serum insulin concentrations increased across fetuin-A quartiles after adjustment for age, sex, community, current smoking, current drinking, BMI (both p for trend <0.0001). HOMA-IR in the fourth (1.41, 95% CI 1.34–1.47), the third quartile (1.37, 95% CI 1.31–1.44) and in the second quartile (1.29, 95% CI 1.23–1.35) was significantly higher as compared with the lowest quartile (1.20, 95% CI 1.14–1.25, p<0.0001, <0.0001, 0.03, respectively, Fig. 1, panel A). Fasting serum insulin concentrations in the highest quartile (6.04 µIU/ml, 95% CI 5.77–6.32), the third quartile (5.95 µIU/ml, 95% CI 5.68–6.23) and the second quartile (5.56 µIU/ml, 95% CI 5.32–5.82) were higher than those in the lowest quartile (5.18 µIU/ml, 95% CI 4.95–5.43, p<0.0001, <0.0001, 0.03, respectively, Fig. 1, panel C). The logistic regression analysis showed that the fetuin-A concentrations were associated with the risk of increased HOMA-IR and elevated fasting serum insulin after adjustment for the full confounders (p for trend 0.0005, 0.009, respectively, Table 3). Among participants with Type 2 diabetes, the similar results about the association of fetuin-A with HOMA-IR and fasting serum insulin concentrations were found (Table 3, Fig. 1, Panel B, D).

**Table 3:** The risk of insulin resistance in relation to each increase of fetuin-A quartile among participants with or without type 2 diabetes.

	Non-type 2 diabetes				Type 2 diabetes		
Fetuin-A quartiles	Model 1	Model 2	Model 3	Fetuin-A quartiles	Model 1	Model 2	Model 3
Insulin resistance							
Q1(≤231.6)	1.00	1.00	1.00	Q1(≤243.5)	1.00	1.00	1.00
Q2(231.6–288.2)	1.29 (1.03–1.61)	1.28 (1.02–1.60)	1.21 (0.95–1.55)	Q2(243.5–307.7)	1.29 (0.98–1.71)	1.31(0.99–1.74)	1.05 (0.76–1.43)
Q3(288.2–359.8)	1.55 (1.24–1.93)	1.54 (1.23–1.92)	1.31 (1.03–1.66)	Q3(307.7–376.2)	1.80 (1.36–2.40)	1.78 (1.34–2.36)	1.46 (1.07–2.00)
Q4(≥359.8)	1.69 (1.36–2.10)	1.72 (1.38–2.14)	1.53 (1.20–1.94)	Q4(≥376.2)	1.87 (1.41–2.48)	1.89 (1.42–2.51)	1.49 (1.09–2.04)
p for trend	<0.0001	<0.0001	0.0005		<0.0001	<0.0001	<0.0001
Elevated fasting serum insulin							
Q1(≤231.6)	1.00	1.00	1.00	Q1(≤243.5)	1.00	1.00	1.00
Q2(231.6–288.2)	1.29 (1.03–1.62)	1.29 (1.02–1.60)	1.21 (0.95–1.55)	Q2(243.5–307.7)	1.13 (0.80–1.58)	1.14 (0.81–1.60)	0.96 (0.67–1.39)
Q3(288.2–359.8)	1.57 (1.27–1.96)	1.57 (1.26–1.96)	1.35 (1.06–1.72)	Q3(307.7–376.2)	1.35 (0.97–1.88)	1.34 (0.96–1.86)	1.10 (0.77–1.58)
Q4(≥359.8)	1.53 (1.23–1.90)	1.55 (1.25–1.94)	1.36 (1.07–1.73)	Q4(≥376.2)	1.81 (1.31–2.50)	1.85 (1.34–2.56)	1.61 (1.13–2.28)
p for trend	<0.0001	<0.0001	0.009		<0.0001	<0.0001	0.004

Data are odds ratios (ORs, 95% confidential interval).

Subjects with equal and/or more than the highest quartile of HOMA-IR index, 2.18 in non-type 2 diabetes group and 4.64 in type 2 diabetes group were defined as having insulin resistance. Subjects with equal and/or more than the highest quartile of fasting serum insulin, 9.4 µIU/ml in non-type 2 diabetes group and 13.9 µIU/m in type 2 diabetes were defined as having high levels of fasting serum insulin.

Model 1, unadjusted;

Model 2, adjusted for age, sex, community, BMI, current smoking, current drinking;

Model 3, adjusted for WHR, blood pressure, TC, LogTG, HDL-C, LDL-C, and LogCRP based on model 2.

## Discussion

To the best of our knowledge, this is the first report on serum feruin-A and type 2 diabetes and insulin resistance in Chinese men and women aged 40 or above. We found that serum fetuin-A concentrations were significantly higher in type 2 diabetic patients than subjects with NGR and IGR. Higher fetuin-A concentrations were independently associated with risk of insulin resistance in not only non-diabetic subjects, but also type 2 diabetic patients.

Epidemiology studies have shown that fetuin-A is associated with incident type 2 diabetes in Americans aged more than 70-years old [17] and in Germans aged 35 to 65 years old [16]. In agreement with previous studies, we demonstrated that increased fetuin-A concentrations were independently associated with the presence of type 2 diabetes in middle aged and elderly Chinese. The European Prospective Investigation into Cancer and Nutrition (EPIC)-Potsdam study [16] found that the association between circulating fetuin-A and type 2 diabetes was modified by the existence of elevated glucose levels. They observed a positive association among participants with elevated plasma glucose levels within the nondiabetic range, whereas fetuin-A was not associated with diabetes risk among participants with normal glucose levels. In our study, we also found that in the participants with IGR, higher fertuin-A concentrations tended to have more odds ratios for prevalence of type 2 diabetes than that in those with NGT. As we know, IGR was epidemic around the world, including in China [20]. It was an important course and risk factor of development of type 2 diabetes. Hence, we should pay more attention to serum fetuin-A concentrations in those with IGR and those with other risk factors for type 2 diabetes. However, we did not find the association of elevated serum fetuin-A with the risk of IGR. Moreover, fetuin-A concentrations were not significantly different between the groups of NGT and IGR. So, we can assume that fetuin-A is not most important during the course of development from NGT to IGR. We found that in our study, serum fetuin-A concentrations were not significantly different between Iso-IFG, Iso-IGT, IFG and IGT group. Studies have demonstrated that IFG and IGT have different pathophysiology [24]. Both IFG and IGT had insulin resistance, but they differ in site of insulin resistance. Individuals with IFG mainly had hepatic insulin resistance and normal muscle insulin sensitivity, while individuals with IGT predominantly were characterized by moderate to severe muscle insulin resistance with normal to slightly reduced hepatic insulin sensitivity. Fetuin-A resulted in insulin resistance through binding insulin receptor tyrosine kinase in adepocytes and skeletal muscle [9]. The elevated serum fetuin-A concentrations might associate with adipose and skeletal muscle insulin resistance other than hepatic insulin resistance. This was consistent to the significant association of serum fetuin-A and insulin resistance indicated as HOMA_IR, which was demonstrated highly correlated with insulin resistance index derived from euglycemic clamp in epidemiology [32].

In our study, we found that fetuin-A was positively correlated with fasting serum insulin and HOMA-IR, the indicators of insulin resistance in type 2 diabetes. It was well known, that insulin resistance is the underlying mechanism of type 2 diabetes [25]. Previous studies demonstrated that feuin-A is a natural inhibitor of the insulin receptor tyrosine kinase in vitro and in rodents [8]–[11], [26], [27]. The rate of autophosphorylation of insulin receptor tyrosine kinase and insulin receptor substrate-1 is decreased in rat liver and skeletal muscle by injection of human recombinant fetuin-A [9]. Fetuin-A knockout mice have improved insulin sensitivity and are resistant to weight gain on a high-fat diet [28], [29]. In humans, high fetuin-A concentrations are associated with insulin sensitivity determined during the euglycemic-hyperinsulinemic clamp or HOMA-IR index in small sample size nondiabetic subjects [12], [13]. We added the evidence that in a large sample size that serum fetuin-A was associated with insulin resistance indicated as elevated HOMA-IR index and fasting serum insulin levels. We found that the association between serum fetuin-A and the risk of type 2 diabetes was no longer statistical significant after adjustment for HOMA-IR. We could assume that insulin resistance was an essential link between fetuin-A and type 2 diabetes. We also found that serum fetuin-A was associated with insulin resistance in the diabetics. It was consistent with what was found in small sample size men in Japanese [30]. However, in Mori's study [13], it was reported that insulin resistance may be not exist in the diabetics, so the significant association between fetuin-A and insulin resistance was not found. However, the diabetics had the higher HOMA-IR and fasting serum insulin than the non-diabetics had in our study. We thought that fetuin-A was associated with insulin resistance unaffected by status of glucose metabolism. It was also reported that fetuin-A can suppress the production of adiponectin, an insulin-sensitizing adipokine [6]. The results suggested that there might be other pathways linked between fetuin-A and insulin resistance. Further studies are needed to explain the mechanisms between fetuin-A and insulin resistance.

We found that fetuin-A concentrations were not significantly associated with serum CRP concentrations in our study. Actually, the association of fetuin-A and CRP was remained controversial [4], [6], [16], [17]. In humans, high serum fetuin-A levels were found to be positively associated with metabolic syndrome and CRP, suggesting that fetuin-A may be causally involved in the pathophysiology of the condition of subclinical inflammation [4], [6]. However, the other study indicated that endogenous fetuin-A can attenuate the inflammatory response [31]. They found a previously unrecognized anti-inflammatory role of fetuin in counter-regulating the innate immune response, and suggest that it may be possible to use fetuin as an experimental anti-inflammatory agent. However, the association of fetuin-A and CRP and the role of fetuin-A in the pathogenesis of low-grade inflammation needs to be studied further.

Limitations were unavoidable. Our study was a cross-sectional design, it could not exactly serve as a causal infer. Further studies are required not only to illustrate the mechanisms of change of fetuin-A concentrations but also to explore the pathogenesis of the association between increased of fetuin-A concentrations and the risk of type 2 diabetes. The insulin resistance was evaluated by HOMA-IR index or serum insulin levels, instead of the hyperinsulinemic-euglycemic clamp. However, Studies show that there is good correlation between estimates of insulin resistance derived from HOMA and from the euglycemic clamp [32].

In conclusion, we found that higher fetuin-A concentrations were independently associated with the risk of type 2 diabetes and insulin resistance in middle aged and elderly Chinese. Insulin resistance played an important role in the association between fetuin-A and type 2 diabetes. Studies are needed to uncover the mechanisms between elevated fetuin-A and insulin resistance and type 2 diabetes.
